# Optogenetic activation of EphB2 receptor in dendrites induced actin polymerization by activating Arg kinase

**DOI:** 10.1242/bio.029900

**Published:** 2017-11-20

**Authors:** Clifford Locke, Kazuya Machida, Yi Wu, Ji Yu

**Affiliations:** Richard D. Berlin Center for Cell Analysis and Modeling, University of Connecticut Health Center, Farmington, CT 06030, USA

**Keywords:** Optogenetics, EphB receptor, Synaptogenesis

## Abstract

Erythropoietin-producing hepatocellular (Eph) receptors regulate a wide array of developmental processes by responding to cell-cell contacts. EphB2 is well-expressed in the brain and known to be important for dendritic spine development, as well as for the maintenance of the synapses, although the mechanisms of these functions have not been fully understood. Here we studied EphB2's functions in hippocampal neurons with an optogenetic approach, which allowed us to specify spatial regions of signal activation and monitor in real-time the consequences of signal activation. We designed and constructed OptoEphB2, a genetically encoded photoactivatable EphB2. Photoactivation of OptoEphB2 in fibroblast cells induced receptor phosphorylation and resulted in cell rounding ­­­­­­– a well-known cellular response to EphB2 activation. In contrast, local activation of OptoEphb2 in dendrites of hippocampal neurons induces rapid actin polymerization, resulting dynamic dendritic filopodial growth. Inhibition of Rac1 and CDC42 did not abolish OptoEphB2-induced actin polymerization. Instead, we identified Abelson tyrosine-protein kinase 2 (Abl2/Arg) as a necessary effector in OptoEphB2-induced filopodia growth in dendrites. These findings provided new mechanistic insight into EphB2's role in neural development and demonstrated the advantage of OptoEphB as a new tool for studying EphB signaling.

## Abstract (precis)

**Summary:** We describe here a new technique to control EphB2 signaling via light and apply this new technique to investigate EphB2's functions in synaptogenesis.

## INTRODUCTION

Erythropoietin-producing hepatocellular (Eph) receptors comprise the largest receptor tyrosine kinase (RTK) family in mammals (Boyd et al., 2014). The 14 known members of the Eph receptor family are further divided into two subfamilies: the nine EphA (A1-A8, A10) receptors which primarily bind to GPI (glycosyl phosphatidylinositol)-linked ephrin-A ligands, and the five EphB (B1-B4, B6) receptors which primarily bind to transmembrane ephrin-B ligands (Janes et al., 2012; Sloniowski and Ethell, 2012). Despite these ligand preferences, multiple studies have shown that cross-subfamily binding is also possible and the Eph receptors' ligand specificities are not absolute (Kullander and Klein, 2002; Himanen et al., 2004; Noberini et al., 2012; Dai et al., 2014). Nevertheless, in most physiologic cases, both the receptors and their ligands are membrane-bound. Thus Eph receptors' signaling initiation *in vivo* typically requires cell-cell contact (Janes et al., 2012; Lisabeth et al., 2013; Boyd et al., 2014). Furthermore, both ephrins and Ephs are capable of transmitting downstream signals into the respective cells presenting them, resulting in the so-called ‘forward’ signaling downstream of the Eph receptors as well as the ‘reverse’ signaling downstream of the ephrins (Janes et al., 2012; Lisabeth et al., 2013). By sensing cell-cell contacts within complex tissue structures, Eph-ephrin interactions regulate a large array of developmental processes such as cell positioning, tissue patterning, axon guidance and synaptogenesis (Sloniowski and Ethell, 2012; Boyd et al., 2014). Dysfunction in Eph/ephrin signaling has also been linked to various pathological processes, such as cancer and Alzheimer's disease (Chen et al., 2012; Boyd et al., 2014).

EphB signaling is important for multiple aspects of neural development. One function is to regulate axon pathfinding during embryonic stage. It is believed that EphB mediates this function by causing growth cone collapse (Pabbisetty et al., 2007; Lin et al., 2008; Schaupp et al., 2014). Meanwhile in dendrite (Bouvier et al., 2008), EphB is believed to regulate spine formation in hippocampal and cortical neurons (Sloniowski and Ethell, 2012). Previous studies have shown that deletion or inhibition of EphBs resulted in reduced spine density and dysmorphic spines in hippocampal neurons (Henkemeyer et al., 2003). Consistently, *in vitro* activation of EphBs by ligands rapidly increased dendritic spine density (Penzes et al., 2003). While these studies established an important role for EphBs in dendritic spine morphogenesis, the molecular mechanisms of these functions are still not fully understood. A current hypothesis is that EphB signaling is initiated at either the dendrite or dendritic filopodia due to contact with innervating axons, which express ephrin ligands; however, the exact effects of local EphB activation on dendritic morphologies have not been defined.

To facilitate further studies of Eph receptors' signaling mechanisms, we sought to develop and characterize better tools to manipulate Eph receptors utilizing optogenetics. The current experimental method for activating Eph receptors relies on the bath application of solubilized ligands, which lacks spatial control and therefore cannot faithfully reproduce endogenous signaling processes that are initiated at subcellular regions of cell-cell contact. In addition, we also seek to overcome the complexity in decoupling consequences of the forward signaling and the backward signaling in the Eph-ephrin interaction, which could be difficult in many systems because the same cells could often express endogenously both the ephrin ligands as well as the Eph receptors.

## RESULTS

### Optically induced optoEphB2 clustering resulted in receptor activation

**Fig. 1.**
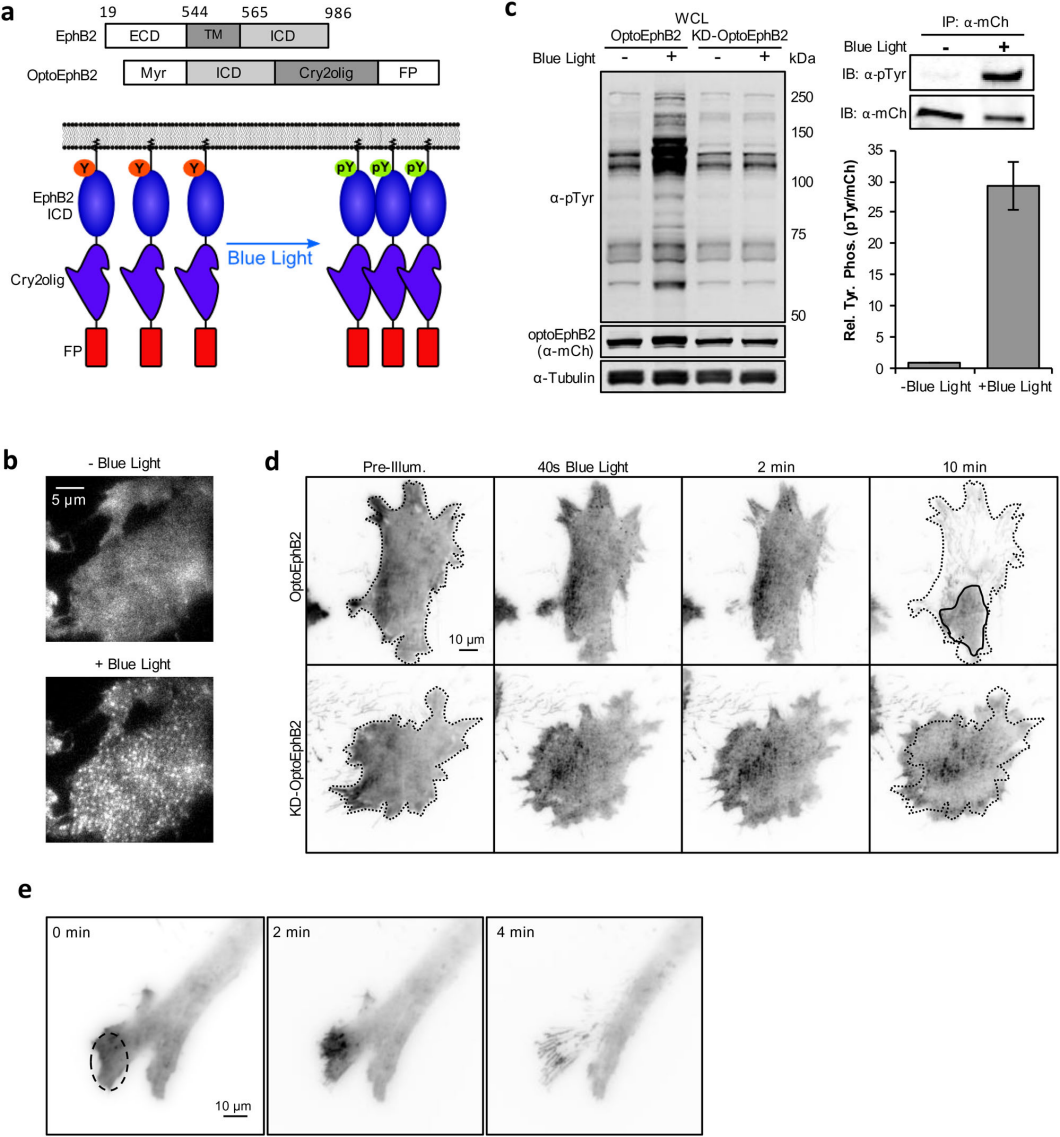
**Optogenetic activation of EphB2.** (A) Schematic illustrations of OptoEphB2 domain structure and the photoactivation process. Blue light illumination induces Cry2 clustering, which results in receptor autophosphorylation (Y, tyrosine; pY, phosphotyrosine) and downstream signaling. ECD, extracellular domain; TM, transmembrane domain; ICD, intracellular domain; Myr, myristoylation signal peptide; FP, fluorescent protein. (B) TIRFM images of optoEphB2-expressing HEK293 cells before and after photoactivation (440 nm, three 250-ms pulses delivered 4.5 s apart), showing optoEphB2 clustering. (C) Left: western blot analysis of whole cell lysates collected from MEFs expressing OptoEphB2-mCherry or KDoptoEphB2-mCherry that were illuminated by blue LED light (∼10^−2^ W/cm^2^), or left in the dark, for 1 min. Right: quantification of OptoEphB2 phosphorylation. Relative tyrosine phosphorylation was assayed in OptoEphB2 immuno-precipitates and quantified by dividing the phosphotyrosine signal by the mCherry signal. Error bars show s.e.m. (*n*=3). (D) Time-lapse TIRF images of MEFs expressing OptoEphB2-mCherry or KD-optoEphB2-mCherry showing kinase dependent cell-rounding after blue light illumination (10 mW/cm^2^, 50-ms pulses at 3 pulses/min). Black dotted lines trace initial cell area and solid line traces the final cell area. (E) Time-lapse fluorescence images of a MEF cell activated by blue light illumination (100-ms pulses, 6 pulses/min) within the specified region of illumination (ROI, black circle). OptoEphB2 clustering and cell process retraction were spatially restricted to the ROI. Time stamps are relative to the start of blue light illumination.

We report the development of OptoEphB2, a genetically-encoded, photoactivatable EphB2 based on the blue light-induced clustering of the *Arabidopsis thaliana* photoreceptor Cryptochrome 2 (Cry2) (Kennedy et al., 2010; Bugaj et al., 2013). The blue light-induced clustering promotes receptor cross-phosphorylation leading to receptor activation (Fig. 1A). This strategy has previously been used to achieve optical activation of FGFR and Trk (Chang et al., 2014; Kim et al., 2014), two other members of the RTK family. However, we found that OptoEphB2 designed using wild-type Cry2 did not yield consistent receptor phosphorylation. We suspected that this is because, unlike most RTKs which only need receptor dimerization for activation, Eph receptors are known to require high-order cluster formation (Davis et al., 1994; Stein et al., 1998), and wild-type Cry2 did not generate clusters that are big enough. Thus a recently identified mutant, Cry2olig (Cry2 E490G), which has a higher tendency to form high-order clusters (Taslimi et al., 2014) was used in our final design. In addition, we replaced the extracellular domain (ECD) and the transmembrane sequence of the EphB2 with an N-terminal myristoylation signal peptide (derived from c-Src) (Fig. 1A,B; Fig. S1). This was done to ensure that only the forward signaling, and not a combination of both the forward and the reverse signaling, is being activated. Conversely, expressing the ECD domains could cause inadvertent receptor activation due to interactions with endogenous ephrins, as well as ECD-mediated receptor-receptor interactions (Himanen et al., 2010). Indeed, constructs that employed full-length Eph receptor sequences failed to localize to the plasma membrane (Fig. S2) in HEK296 cells, and were found mainly on intracellular vesicles, suggesting receptor activation and internalization.

The design of OptoEphB2 shown in Fig. 1 resulted in plasma membrane localization and robust blue light-induced clustering (Fig. 1B). OptoEphB2-expressing mouse embryonic fibroblasts (MEFs) subjected to blue LED light illumination exhibited significantly higher overall tyrosine phosphorylation compared to cells left in the dark (Fig. 1C). In contrast, blue light produced no increase in tyrosine phosphorylation in cells expressing kinase-dead optoEphB2 (KD-optoEphB2), which contained a mutation (K99M in OptoEphB2) in the kinase domain (Fig. 1C). The most significant increase in phosphorylation was observed near 135 kDa, consistent with the size of OptoEphB2. Anti-phosphotyrosine blot analysis of immune-precipitated OptoEphB2 indeed verified this and showed an approximately 29-fold increase of tyrosine phosphorylation (Fig. 1C) in blue light-illuminated samples.

To further test whether OptoEphB2 phosphorylation serves a functional role, we examined if photoactivation of OptoEphB2 produced the same cellular phenotypes as those caused by ligand-mediated activation. One of the most widely reported cell phenotype upon Eph activation is retraction of cellular protrusions and cell rounding (Zou et al., 1999; Elowe et al., 2001; Zimmer et al., 2003; Lin et al., 2008; Lisabeth et al., 2013). We first verified that the MEF cell line indeed has such as response when stimulated with pre-clustered ephrinB1-Fc ligands (Fig. S3). Consistently, we found that photoactivation of OptoEphB2 in MEFs also quickly induced cell rounding (Fig. 1D; Movie 1). As expected, the observed cell rounding phenotypes were kinase-dependent, as blue light illumination of KD-optoEphB2 produced receptor clusters but did not result in significant reduction in cell area (Fig. 1D). Furthermore, when photoactivation is restricted to sub-cellular regions using digital light patterning (Guo et al., 2009), both receptor clustering and cell retraction were spatially restricted to the photoactivated regions, while non-illuminated regions were unaffected (Fig. 1E), demonstrating the ability of spatially controlling EphB signaling with OptoEphB2.

### Kinetics of OptoEphB activation

**Fig. 2.**
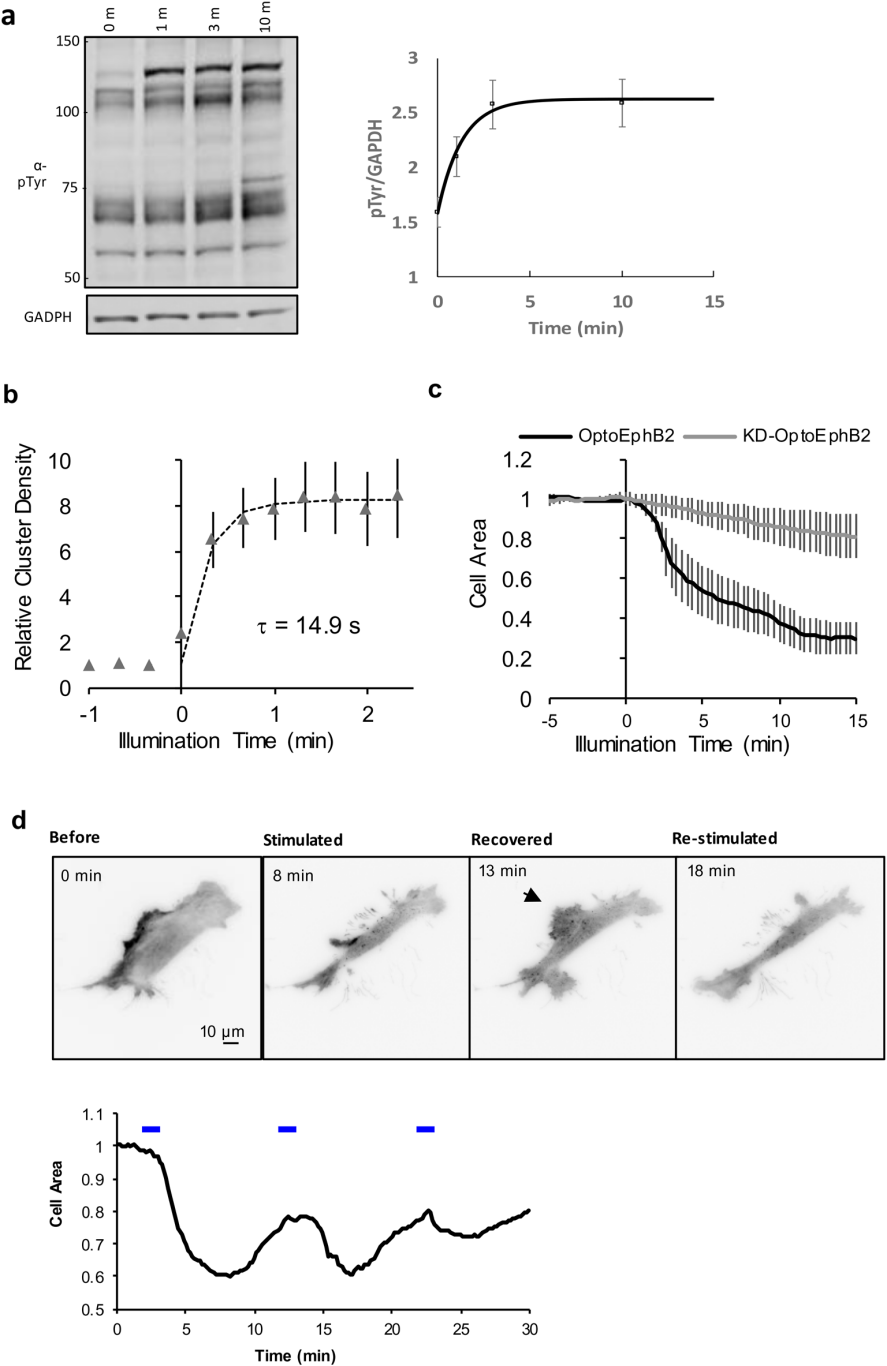
**Kinetics of OptoEphB2 activation.** (A) Left: western blot (pTyr) of MEF cell lysates after specified time of blue light illumination. Right: quantification of normalized total pTyr immunoactivity in cell lysates (*n*=3). Solid line denotes exponential fit with a time constant of 49.7 s. (B) OptoEphB2 cluster density (# of cluster/cell area, *n*=4) in MEFs under blue light activation. The dotted line denotes exponential fit with a time constant of ∼15 s. (C) Quantification of MEF cell rounding kinetics. Cell area was normalized to the mean value prior to photoactivation. Both cells expressing OptoEphB2 (black line, *n*=6) and the control cells expressing Kinase-dead mutant (gray line, *n*=7) were show for comparison. (D) Testing of the reversibility of OptoEphB2-induced cell rounding. MEFs expressing OptoEphB2 were stimulated with multiple trains of blue light (6×100-ms pulses, 0.1 Hz), while cell morphology was monitored with TIRFM. Top panel shows selected frames in time-lapse data, showing MEF contraction and recovery after the first round of stimulations, as well as the re-contraction after the second round of stimulations. Bottom panel shows the quantification cell area over time, normalized to the average cell area prior to the first stimulation (2 min). Blue bars denote time for optical stimulation. All error bars denote s.e.m.

To characterize the kinetics of OptoEphB2 activation (Fig. 2), we analyzed the rate of tyrosine phosphorylation (Fig. 2A) as well as cluster formation in cells with live cell time-lapse imaging (Fig. 2B). We found that the OptoEphB2 cluster density increases quickly with a time constant of ∼15 s, when cells were illuminated with 440-nm blue LED (∼10 mW/cm^2^, 0.1 Hz pulsed). In comparison, western blot analysis of receptor phosphorylation (Fig. 2A) in cell lysates collected at various time delays after blue light illumination (∼0.5 mW/cm^2^ blue LED) showed a similarly rapid increase, with a time constant of ∼50 s. Saturation of phosphorylation was reached at about ∼3 min and the phosphorylation level remained constant until the end of the experiment (10 min). The kinetics of the cell rounding after OptoEphB2 activation was also quantified by measuring cell area in time-lapse microscopy data (Fig. 2C). As expected, the kinetics of cell rounding are significantly slower than the receptor phosphorylation and cluster formation, as the signal propagates from the receptor to the downstream effectors that ultimately remodel actin cytoskeleton and cell morphology. Collapse of membrane cell protrusions was observed with only a delay of 1-2 min, and cell rounding typically takes ∼10 min to finish.

A previous report has shown that Cry2olig cluster formation is reversible (Taslimi et al., 2014). Thus, we tested if the OptoEphB2-induced cell phenotype was also reversible. By leaving a pre-stimulated cell in the dark, we observed that the receptor clusters dissipated within 5 min (Fig. 2D; Movie 2). This is somewhat surprising because the dissipation rate is faster than previously reported for cytoplasmic Cry2olig-mCherry (Taslimi et al., 2014). This may be due to differences in clustering on the plasma membrane versus cytosol or the effects of EphB2 ICD signaling. Additionally, the cell started to re-expand by generating highly dynamic membrane protrusions (Fig. 2D; Movie 2), indicating that the whole process was reversible. In addition, after recovery of the cell area, we could reinitiate the cell rounding process again by a new round of blue light illumination, indicating that the OptoEphB2 receptor activation together with its downstream processes are indeed reversible.

### OptoEphB2 activation in dendrites of hippocampal neurons induced actin nucleation

**Fig. 3.**
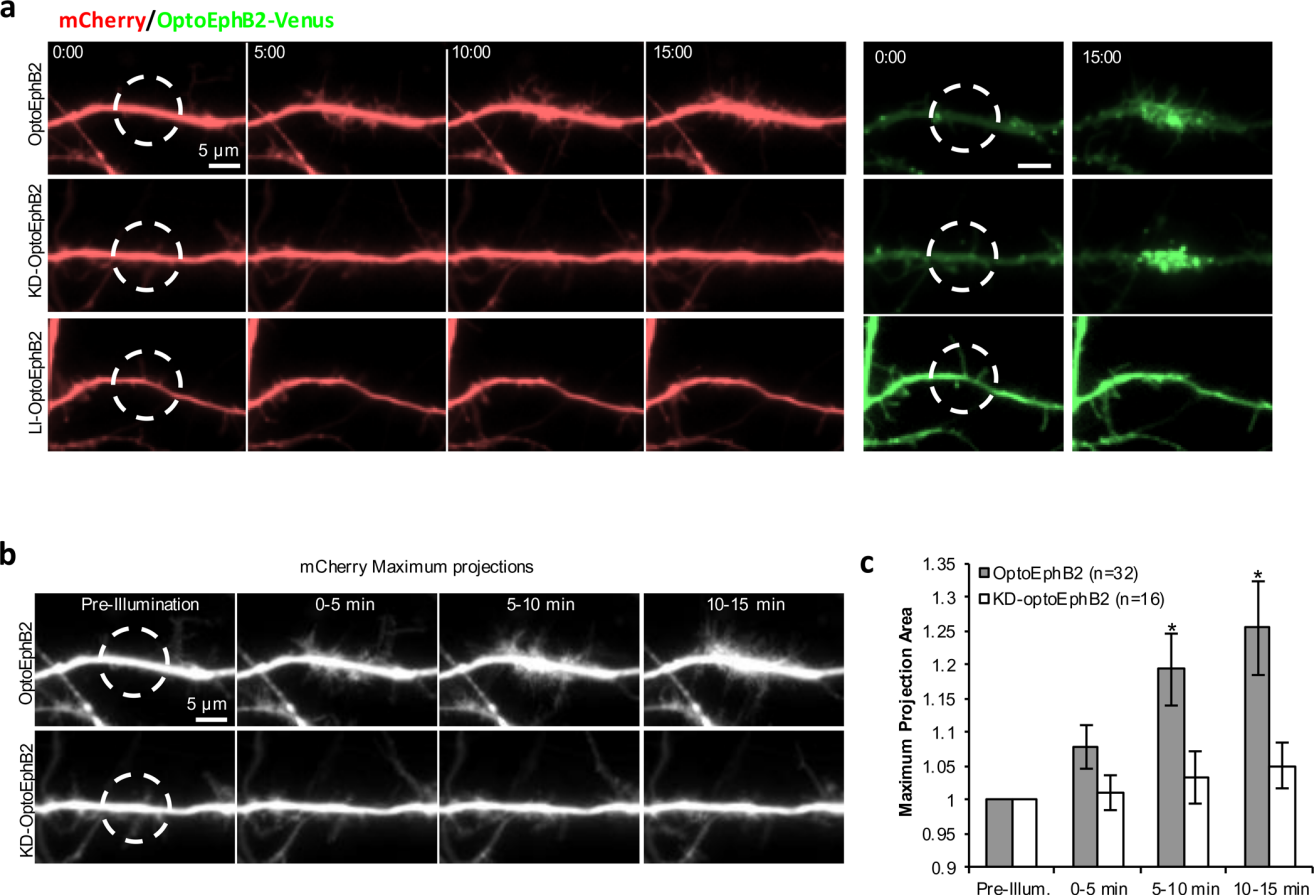
**OptoEphB2 activation in dendrites induces dynamic filopodial protrusions.** (A) Time-lapse images of neurons expressing OptoEphB2-Venus, or its mutant variants, and mCherry (as a volume marker). Cells were photo-activated via blue light illumination (50-ms pulses, 3 pulses/min) over the indicated ROI (white circles) in dendritic segments. Images were mCherry fluorescence. Time labels are relative to the start of photoactivation. (B) Images of maximum-intensity projection over time (5-min durations) from the dendritic segments shown in A. (C) Quantification of increased filopodial protrusions. Dendritic areas within the ROI were measured from maximum intensity projections and normalized to measurements before photoactivation. Error bars are s.e.m. (*n*=32 for OptoEphB2, *n*=16 for KD-optoEphB2). *P<0.05; *t*-test, comparing OptoEphB2 to KD-optoEphB2.

We first tested whether OptoEphB2 expression alters endogenous EphB2 localization in hippocampal neurons (Fig. S6). EphB2 was detected primarily in soma-dendritic compartments and appears to be slightly clustered along the dendritic shaft (Fig. S6). We detected no significant changes in this localization pattern in cells expressing OptoEphB2-mCherry (Fig. S6). Next, we performed OptoEphB2 activation in primary hippocampal neurons of DIV9-11. We found that localized photoactivation of OptoEphB2 induced formation of dynamic filopodia-like protrusions within the region of activation (Fig. 3A,B; Movie 3). The results were compared with cells expressing KD-optoEphB2 or LI-optoEphB2, an OptoEphB2 mutant that carries a D387A change in the Cry2 sequence, making the molecule light-insensitive (LI). Clusters were produced from OptoEphB2 or KD-optoEphB2, but not LI-optoEphB2 (Fig. 3A); however, OptoEphB2 cells produced filopodial protrusions. Thus, the observed effect requires both clustering and the kinase activity. Filopodia appeared and disappeared transiently during the experiment. To quantify these dynamic morphological changes in dendrites, we generated maximum intensity projection images by always taking the brightest pixels over 5-min time-lapse segments (Fig. 3B). Thus, the cell area in the final image represented the total area filopodia explored during their dynamic protrusion/retraction process. We measured cell areas of the maximum intensity projection images and performed multivariate linear regression analyses against stimulation time. We found that that cells expressing OptoEphB2, but not KD-optoEphB2, exhibit significant increased cell area after activation (*P*=0.008). Furthermore, after 5-min photoactivation, *t*-test showed significant differences in cell areas between OptoEphB2 cells and KD-optoEphb2 cells (Fig. 3C).

**Fig. 4.**
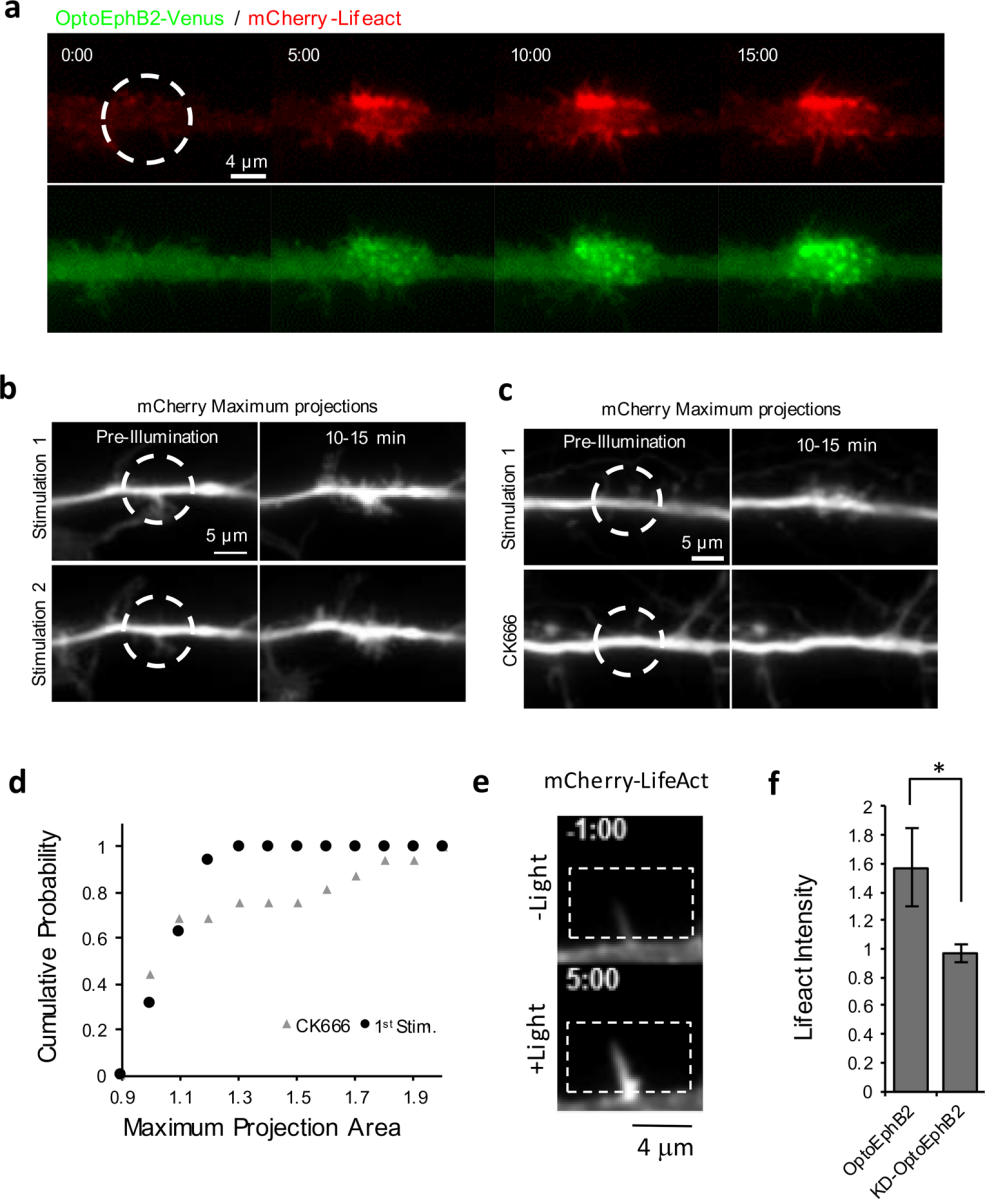
**OptoEphB2 activation in dendrites induces actin polymerization.** (A) Time-lapse images of mCherry-Lifeact in the ROI during photoactivation of OptoEphB2. (B) Maximum-intensity projection images of a dendrite undergoing two rounds of OptoEphB2 photo-activation. Neural cells (DIV11) expressing OptoEphB2-Venus and mCherry (shown) were photoactivated over the indicated ROI (dash line). Two rounds of photoactivation were spaced with 20 min of incubation in the dark. (C) Same as in B except the second round of photoactivation was carried out with the presence of CK666 (200 μM). (D) The Kolmogorov–Smirnov plot showing the cumulative probability of the cell area increase (based on maximum intensity projection, *n* =16 each) from the two-round activation protocol shown in B. (E) Images of mCherry-Lifeact in a dendritic filopodium before (-1:00) and after (8:00) OptoEphB2 photoactivation. (F) Quantification of Lifeact intensity in filopodia. Normalized intensity was calculated cell-by-cell by averaging the intensity in all filopodia (>5 per cell) along the ROIs before and after illumination, normalizing to the pre-illumination value, and averaging between cells. Error bars, s.e.m. (*n*=11 cells for optoEphB2, *n*=9 cells for KD-optoEphB2). *P<0.05; *t*-test.

Dendritic filopodia are membrane protrusions supported by actin cytoskeleton (Korobova and Svitkina, 2010). To see if OptoEphB2-induced filopodia are indeed a result of actin polymerization, we labeled F-actin by transfecting cells with mCherry-Lifeact construct (Addgene plasmid #54491). We found that after photo-activation of OptoEphB2, Lifeact accumulated in a punctate distribution on the periphery of the dendrite in the activation region of illumination (ROI) (Fig. 4A), indicating increased actin polymerization at the base of newly formed filopodia. To further examine this phenomenon, we designed a double-activation protocol to test whether the effects were sensitive to CK666, an Arp2/3-dependent actin nucleation inhibitor. The protocol was based on the earlier finding that OptoEphB2 signal is reversible (Fig. 2D). Similarly, OptoEphB2-induced filopodia retract after samples sit in the dark for 20 min, allowing a second round of photoactivation at the same dendritic region, which produces the dynamic filopodia again (Fig. 4B). To test effect of CK666, we treated the cell with CK666 right before the second stimulation and compared the results with the first stimulation (Fig. 4C). Indeed, we found (Fig. 4D) that the induction of filopodia can be blocked by treatment of CK666 (Fig. 4C,D), suggesting that EphB activation induces branched actin nucleation, which in turn gives rise to increased F-actin and formation of new filopodia.

It has been suggested that axon-dendritic contact during neural development leads to EphB activation directly in dendritic filopodia (Sloniowski and Ethell, 2012). Therefore, we further tested whether OptoEphB2 activation can induce actin polymerization specifically in dendritic filopodia. The experiments were carried out in primary hippocampal neurons of DIV9-11 co-expressing OptoEphB2-Venus and mCherry-Lifeact. Filopodia were stimulated by targeting blue light to a ROI oriented lengthwise along, but offset from, the dendritic shaft. We found that indeed OptoEphB2 activation resulted in significantly increased Lifeact signal (∼57% increase) in dendritic filopodia (Fig. 4E,F), consistent with the idea that the stimulation promotes F-actin nucleation just like in dendritic shafts.

### Arg kinase acts downstream of EphB2 and is required for OptoEphB2-induced dendritic actin polymerization

**Fig. 5.**
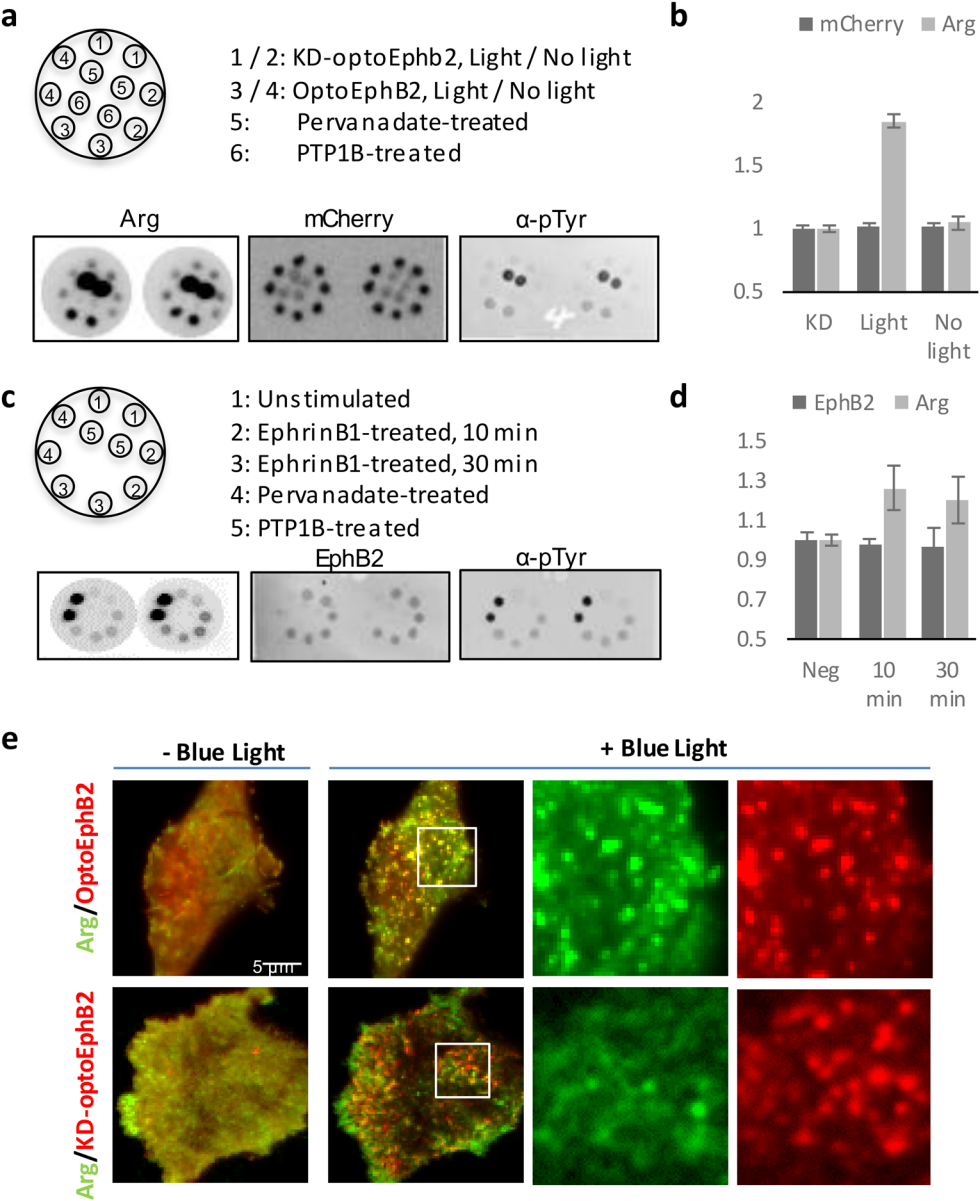
**OptoEphB2 interacts with Arg.** (A) Rosette assay of whole cell lysates (MEFs expressing OptoEphB2-mCherry or KD-optoEphB2-mCherry) probed with a panel of purified SH2 probes (only Arg-SH2 is shown) as well as anti-mCherry and anti-pTyr. The top graph illustrates the lysates spotting pattern. Pervanadate- and PTP1B-treated samples were spotted at half volume and serves as positive and negative controls, respectively. (B) Quantification of Arg-SH2 and anti-mCherry (represent total OptoEphB2-mCherry expression) bindings to cell lysates. KD, KD-optoEphb2 lysate (with light stimulation). (C) Same as in A except lysates are from cells stimulated with ephrinB1-Fc ligand. Lysates were probed with Arg-SH2, anti-EphB2 and anti-pTyr. (D) Quantification of Arg-SH2 and anti-EphB2 bindings to cell lysates shown in C. (E) Fluorescence images of MEF cells co-expressing Arg-YFP and OptoEphB2-mCherry showing light-induced co-clustering of Arg and OptoEphb2, but not with KD-optoEphB2. Error bars are s.e.m.

To elucidate the molecular mechanism of OptoEphB2-induced actin polymerization in dendrites, we performed a high-throughput phosphotyrosine profiling assay previously described (Machida et al., 2007) and dubbed as the ‘Rosette’ assay. The Rosette assay is a reverse-phase protein-binding assay using a library of purified Src-homology 2 (SH2) proteins as probes to detect interaction with a small amount of cell lysate spotted on membrane. Because SH2 domains are the phosphotyrosine-binding modules of many important RTK effectors, the assay allows identification of potential SH2-containing effectors by examining changes in their binding to cell lysates. Using a library of 48 probes, we performed the screening on lysates from cells stimulated with OptoEphB2 or KD-optoEphB2 (Fig. 5A,B; Fig. S4). For comparison, a similar assay is also performed on cells expressing wt-EphB2 stimulated with ephrinB1 ligand (Fig. 5C,D; Fig. S5). In both cases, we found that the SH2 probe from Arg, a non-receptor tyrosine kinase, exhibited highest induced binding to activated lysates. To further validate the interaction between EphB2 and Arg, we performed a co-clustering experiment in cells co-expressing Arg and OptoEphB2 (Fig. 5E). Using total internal reflection fluorescence microscopy (TIRFM), we found that Arg molecules cluster spontaneously on cell membrane. However, after blue-light photoactivation, the number of Arg clusters increased significantly and co-localized with the light-induced OptoEphB2 clusters (Fig. 5E), consistent with the hypothesis that OptoEphB2 interacts with Arg. As expected, the co-clustering was not observed in KD-optoEphB2 cells (Fig. 5E).

**Fig. 6.**
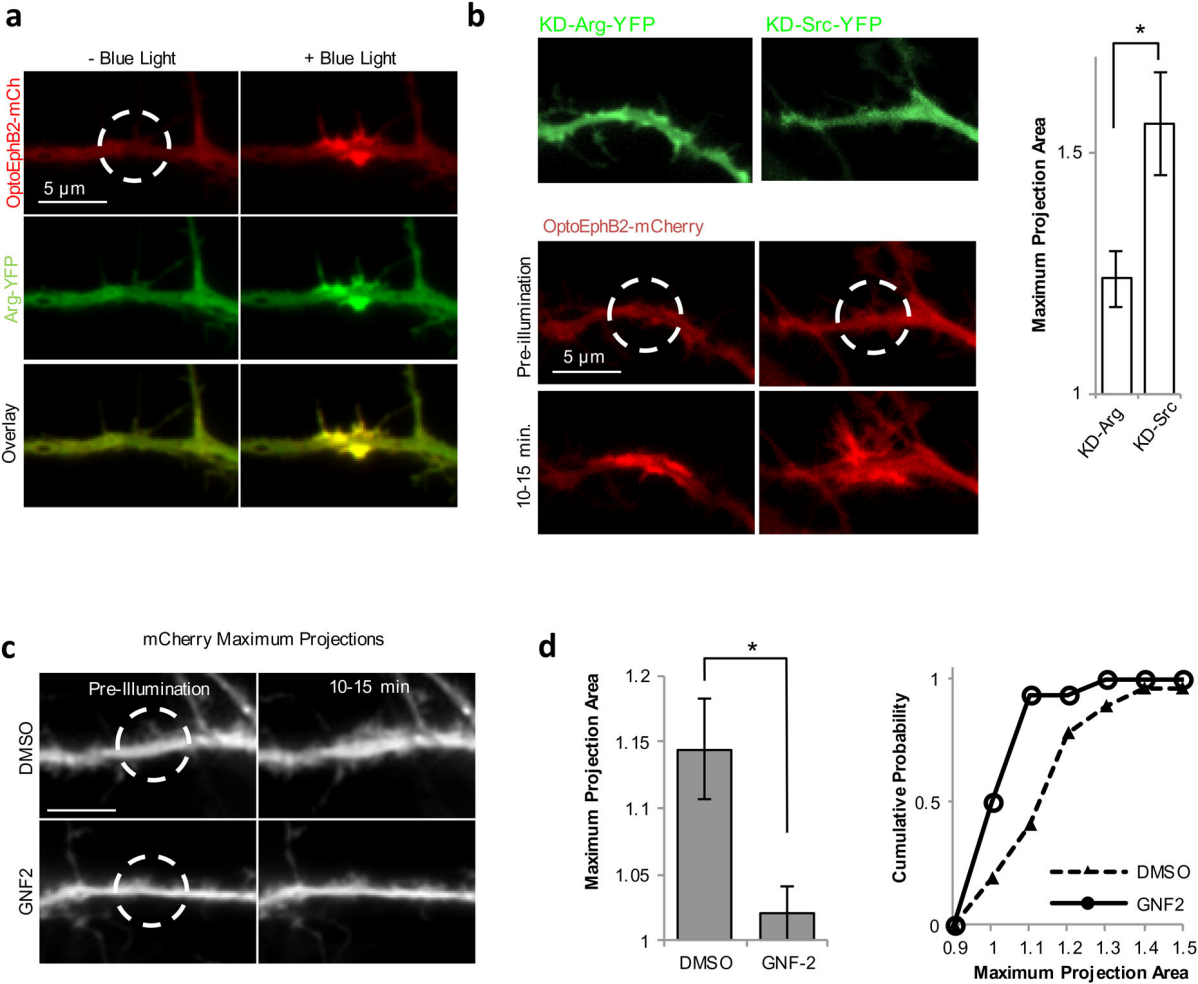
**OptoEphB2-induced dendritic filopodia requires Arg kinase.** (A) Blue light induced co-clustering of OptoEphB2 and Arg in dendrites in neuron cells (DV11) expressing OptoEphB2-mCherry and Arg-YFP. (B) Left: images of maximum-intensity projection from neural cells (DIV11) co-expressing OptoEphB2-mCherry and KD-Arg (top) or KD-Src (bottom). Blue-light induced clustering of OptoEphB2 but no filopodial growth in KD-Arg cells. The inhibitory effect was not observed with KD-Src expression. Right: quantification of cell area increase (*n*=11, based on maximum-intensity projection) in the presence of KD-Arg or KD-Src. (C) Images of maximum-intensity projection from neural cells (DIV 11) co-expressing OptoEphB2-Venus and mCherry. Cells were either treated with GNF2, a specific Arg inhibitor, or DMSO, as controls. The images showed that GNF2 treatment, but not DMSO treatment, abolished OptoEphB2 induced dendritic filopodia growth. (D) Quantification of the effects of GNF2 treatment (*n*=16) in comparison to DMSO (*n*=27). Light-induced dendritic area increases were quantified with maximum-intensity projection images (as shown in C). The left panel showed the average values and the right panels showed the cumulative distributions. *P<0.05; *t*-test. Error bars are s.e.m.

Arg is a regulator of actin cytoskeleton. It has been shown to play a critical role in processes such as cell protrusion (Lapetina et al., 2009) and dorsal-wave formation (Boyle et al., 2007). In neurons, Arg has been shown to be important for the maintenance of cytoskeleton stability in the dendritic spines (Lin et al., 2013). Thus, we suspect Arg acted downstream of OptoEphB2 to promote dendritic actin polymerization. To test this hypothesis, we co-expressed Arg and OptoEphB2 in hippocampal neuron and found that photoactivation of OptoEphB2 resulted in its co-clustering with Arg (Fig. 6A) in dendrites, suggesting interactions between the two in dendrites. When kinase-dead Arg (KD-Arg) mutant was expressed, OptoEphB2 still cluster normally in response to light (Fig. 6B); however, the KD-Arg inhibited the filopodia-inducing effects of OptoEphB2 (Fig. 6B; Movie 4). Importantly, such inhibitory effect is specific to Arg, as expression of kinase-dead Src (KD-Src), which is also a non-receptor tyrosine kinase with an SH2 domain, did not exhibit such effects (Fig. 6C). Similarly, we also found that the filopodia-inducing effects of OptoEphB2 are also abolished in the presence GNF2, a chemical inhibitor specific to the Abl/Arg family kinases (Fig. 6C,D). Combined, these results indicated that Arg is a required effector in the EphB2 pathway to promote actin polymerization.

### OptoEphB2 induced dendritic filopodia without activating Rac1 and CDC42

**Fig. 7.**
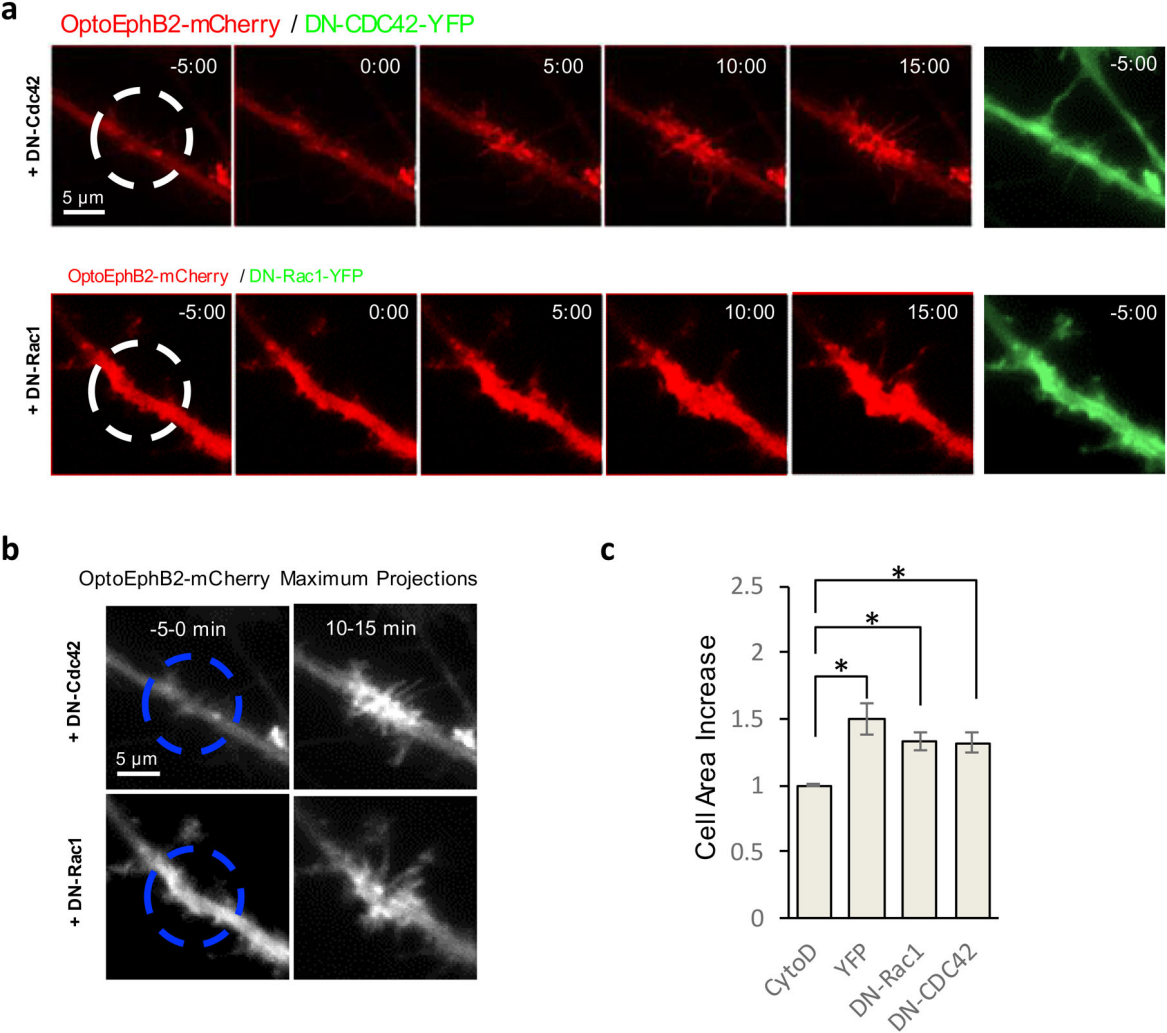
**OptoEphB2 can induced dendritic filopodia growth without activating Rac1 and CDC42.** (A) Time-lapse fluorescence images of neural cell (DIV 10-11) expressing OptoEphB2-mCherry and dominant negative CDC42 (DN-CDC42-YFP, top) or dominant negative Rac1 (DN-Rac1-YFP, bottom). Fluorescence signals were from OptoEphB2. (B) Images of maximum-intensity projection before and after photoactivation (10-15 min) from cells shown in A. (C) Quantifications of cell area increases (based on maximum-intensity projection) for cells expressing DN-Rac1-YFP (*n*=15), DN-CDC42-YFP (*n*=13) and YFP only (*n*=11). Results were compared to control dataset of cells treated with actin polymerization inhibitor Cytochalasin D (*n*=4). *P<0.05; *t*-test. Error bars are s.e.m.

Previous studies of EphB signaling have identified several RhoGEFs, including those of Rac1 and CDC42, that can bind to EphB (Penzes et al., 2003; Irie and Yamaguchi, 2002). Both Rac1 and CDC42 are central regulators of actin cytoskeleton, and therefore could play important roles in OptoEphB2-induced dendritic actin polymerization. On the other hand, existing literature on Arg suggested that Arg regulates actin cytoskeleton directly by activating actin nucleation promoters, such as cortactin (Boyle et al., 2007), or Nck (Antoku et al., 2008). Thus, its action may be independent of the RhoGTPases activation in dendrites. To see if this is true, we further performed OptoEphB2 photoactivation experiment while inhibiting either Rac1 or CDC42 by overexpressing dominant-negative (T17N) Rac1 (DN-Rac1) or dominant-negative (T17N) CDC42 (DN-CDC42). We found that neither condition resulted in strong inhibition of OptoEphB2-induced filopodial growth (Fig. 7). Subtle effects on filopodia morphology can be observed (Fig. 7A). For example, expressing DN-Rac1 seems to result in formation of longer filopodia after OptoEphB2 activation. Nevertheless, induction of filopodia was observed in all cells tested after OptoEphB2 activation. The results suggested that Arg-dependent mechanism for inducing filopodia is independent of the RhoGTPases pathway downstream of EphB.

## DISCUSSION

Despite extensive research into the mechanisms of Eph-ephrin signaling, the system has often defied efforts of establishing a unified signaling pathway to explain Eph receptor functions. The complexity of the system is manifested, in part, from the multitude of cellular responses observed from the signal activation, many of which seem to be apparently contradictory of each other. For example, Eph-ephrin signaling was found to be capable of both suppressing (Miao et al., 2001) and activating (Genander et al., 2009) proliferation, and both strengthening (Carter et al., 2002) and weakening (Miao et al., 2000) focal adhesions. While Eph genes are consistently implicated in various forms of cancers (Pasquale, 2010), they can serve both as cancer suppressor (Batlle et al., 2005) and as promoter (Fang et al., 2005). In our study, this complexity was again demonstrated in that stimulation of OptoEphB2 have vastly different morphological consequences in fibroblasts (cell rounding) and in neurons (filopodial protrusion).

The complexity of the EphB pathway demands more versatile research tools. Here we demonstrated a new optogenetic construct for activating Eph receptor signaling. It is worthwhile to discuss the key advantages, as well as potential problems, of the optogenetic method in comparison to ligand-mediated activation. (i) The main advantage of the optogenetic method is accurate spatial and temporal control over Eph receptor signaling. This specific feature carries particular importance for Ephs, whose *in vivo* activation is, by definition, spatially and temporally confined due to the nature of cell-cell contact. (ii) We also demonstrated that OptoEphB activation is reversible, whereas ligand activation is essentially irreversible. This reversibility is useful for experiments that require repeated cycles of activation in a controlled experimental setting, as we have demonstrated in this paper. (iii) Finally, although not directly utilized in the current study, OptoEphB presumably allows for testing the biological functions of specific members among the Eph family or, by extension, the effect of co-clustering two or more Eph receptors in a precise manner. Ligand-mediated stimulation does not allow control over cluster composition, since ligand binding may be promiscuous (Kullander and Klein, 2002; Himanen et al., 2004; Noberini et al., 2012; Dai et al., 2014) and receptor-receptor binding interfaces allow interactions between Ephs of different types (Himanen et al., 2010; Janes et al., 2011, 2012), thus any ligand may result in activation of multiple receptor subtypes. On the other hand, a potential disadvantage is that OptoEphB2 requires ectopic expression, which may perturb cell physiology. Eph receptors are also known to have ligand-independent and kinase-independent signaling activities (Genander et al., 2009; Himanen et al., 2010; Seiradake et al., 2010; Boyd et al., 2014). Whether these activities are perturbed by OptoEphB expression has not been fully investigated yet.

Our results also provided new insights into EphB signaling in synaptogenesis and dendritic spine development. While it is not entirely surprising that EphB2 plays a role in regulating actin cytoskeleton, previous studies have been largely focused on EphB2 signaling onto the RhoGTPase pathway, e.g by activating Rac1 or CDC42 (Penzes et al., 2003; Irie and Yamaguchi, 2002), and the importance of the Arg activation in this signaling pathway has not been previously identified. Furthermore, our data indicated that Arg could activate dendritic actin polymerization independent of the RhoGTPase activation. While this is consistent with the current literature on Arg's biochemical functions, it is interesting to note that previous experiments have found that inhibiting RhoGTPases Rac1 could block EphrinB-induced synaptic spine formation (Penzes et al., 2003). A possible explanation is that Rac1 is needed for the reorganization of the presynaptic structure, which is a part of the synaptogenesis process; while our experiments focused entirely on the dendritic region.

The importance for EphB2 in dendritic spine morphogenesis had been well accepted, but the exact mechanism has been under debate. Comparison between EphB1/B3 double-knockout and EphB TKO neurons suggested that EphB2 enhances the motility of dendritic filopodia, thereby increasing the probability of axo-dendritic contact formation (Kayser et al., 2008). However, one study of ligand-mediated stimulation of neurons suggested that EphB signaling shortens and stabilizes existing dendritic filopodia (Moeller et al., 2006), a dynamic change thought to initiate a transition from dendritic filopodia to dendritic spines (Ziv and Smith, 1996). Our experiments provided a real-time view of the cellular response from EphB2 activation, and provided unambiguous evidence that increased F-actin accumulation, as opposed to the stabilization of the F-actin, is the key consequence here. It also seems that EphB2 signaling may serve as a positive feedback mechanism in spine formation by promoting a high density of dendritic filopodia near sites of axo-dendritic contact, which would lead to more local axo-dendritic contacts.

## MATERIALS AND METHODS

### Reagents and cell culture

All primary antibodies used in the study were purchased from commercial sources, including anti-phosphotyrosine (Cell Signaling, Danvers, MA), anti-tubulin (Thermo Scientific, Waltham, MA), anti-mCherry (Thermo Scientific, Waltham, MA), and anti-EphB2 (Santa Cruz Biotechnology, Dallas, TX). IRDye 680- and IRDye 800-labeled secondary antibodies were purchased from LI-COR (Lincoln, NE). All other chemicals used for the experiments were purchased from Sigma-Aldrich (St Louis, MO).

OptoEphB2 as well as all variants and control constructs were derived from CRY2olig-mCherry or CRY2oligPHR-mCherry plasmids (Kennedy et al., 2010; Taslimi et al., 2014) obtained from Addgene (Cambridge, MA). A gateway cassette (ThermoFisher, Watham, MA) was cloned into the multiple cloning regions of these vectors to convert them into gateway vectors. The intracellular domain (aa 565- 986) of EphB2, together with an N-term myristoylation signal peptide (MGSNKSKPK) was amplified and cloned into gateway entry vector pDONR223 (ThermoFisher). The final optoEphB2 construct was obtained via LR recombination of the entry clone with a CRY2oligPHR-mCherry-derived gateway vector. For two-color microscopy experiments, an mVenus labeled variant (OptoEphB2-Venus) was often used in place of the mCherry labeled one (OptoEphB2-mCherry), because it was detected that long exposure of green light could potentially activate OptoEphB2 to some degree. Using OptoEphB2-Venus can help reducing the total exposure of cells to green excitation in some experiments. OptoEphB2-Venus was constructed by replacing mCherry in OptoEphB2-mCherry via standard PCR cloning method. All other related variants were constructed in a similar manner. The kinase-dead KD-optoEphB2 construct was made by site-directed mutagenesis to introduce a K99M mutation. The light-insensitive Cry2 mutant, containing a D387A mutation, was previously described (Bugaj et al., 2013), and the mutation was introduced by subcloning fragments that contained the mutation. Both Arg and KD-Arg constructs were gifts from the Koleske lab (Yale University). The mCherry-Lifeact plasmid (Addgene plasmid #54491) was a gift from Michael Davidson (University of Florida).

To obtain cell lines expressing OptoEphB2, the complete OptoEphB2-mCherry coding sequence was subcloned into a lentiviral vector pLIX401 (Addgene plasmid # 41390), a gift from David Root (Broad Institute). Viral particles produced from HEK293FT cells (ThermoFisher) were used to infect MEF-TetOff cells (Clontech, Mountain View, CA). All cell lines used were maintained in Dulbecco's Modified Eagle Medium (Lonza, Switzerland) with fetal bovine serum (BioWest, Kansas City, MO). Cell lines were from commercial sources and had not been independently authenticated in the lab. Primary hippocampal neurons were plated and maintained as previously described (Tatavarty et al., 2012). Cells were isolated from pre-dissected embryonic hippocampi (E17-19) of Sprague-Dawley rats (Brainbits Inc, Springfield, IL) and plated onto plasma-cleaned 30-mm coverslips coated with 0.05% poly-L-lysine at 90,000-100,000 cells/dish. Transient transfections of neurons were carried out using Lipofectamine 2000 (ThermoFisher), following manufacturer's protocol with some modification 1-2 days before imaging.

### Biochemical assays

For western assays, cells were lysed in modified kinase lysis buffer (KLB) (Ditlev et al., 2012) as previously described, with 0.1% SDS added to aid in solubilizing large OptoEphB2 or optoEphB2-KD clusters. SH2 domain rosette screening was performed as described previously (Machida et al., 2007). Briefly, lysates were diluted with 2× spotting solution (100 mM Tris–HCl, pH 6.8, 30% glycerol, 2% SDS) to approximately 4 μg/μl and spotted in duplicate in a rosette pattern on nitrocellulose membranes. Membranes were blocked with 5% milk in TBST [25 mM Tris– HCl, pH 8.0, 150 mM NaCl, and 0.05% (v/v) Tween-20] and incubated with 200 nM GST-SH2 domains labeled with GSH-HRP for 2 h in a 96-well chamber plate. Each well was washed with TBST and chemiluminescent detection and quantification was performed using Carestream Image station system and Carestream MI software.

### Microscopy and image analysis

#### Live cell imaging and optogeneic control of OptoEphB2

Most live cell imaging experiments were carried out on a Nikon (Tokyo, Japan) Ti-E inverted fluorescence microscope with a 60× TIRF objective (NA=1.49, Nikon). Images were acquired with an iXon Ultra EM-CCD (Andor, Oxford Instruments, Abingdon, Oxfordshire, UK). The microscope was placed within a temperature-regulated imaging chamber and cells were maintained at 37°C during imaging. For imaging mammalian cell lines, cells were kept in DMEM/F12 containing 2% FBS and 20 mM HEPES. For imaging neurons, the cells were kept in imaging medium containing 117 mM NaCl, 5 mM KCl, 1.25 mM NaH_2_PO_4_, 20 mM HEPES, 50 mM dextrose, 1 mM MgCl_2_, 2 mM CaCl_2_, and 100 mg/L BSA. Co-clustering assay was performed on an Olympus (Tokyo, Japan) IX81 TIRF microscope equipped with a 60× TIRF objective (NA=1.49, Olympus) and a TE-cooled EM-CCD (PhotonMax, Princeton Instruments, Trenton, NJ). The 488-nm line of an argon ion laser was used to excite GFP and to photoactivate OptoEphB2, a 562-nm DPSS laser was used to excite mCherry, and a 442-nm DPSS laser was alternatively used for photo-activation of optoEphB2. Spatial control of OptoEphB2 was achieved using a Mosaic illumination system (Andor) coupled to a 440-nm LED (CoolLED, Andover, Hampshire, UK) on the Nikon Ti-E microscope, unless otherwise noted. For focal illumination of dendrites, the mosaic was used to deliver blue light to a 40-pixel-diameter circular region. The ROI was expanded to cover the whole mosaic for global illumination. All image quantifications and analyses were performed in ImageJ (NIH).

## Supplementary Material

Supplementary information

## References

[BIO029900C1] Antoku S., Saksela K., Rivera G. M. and Mayer B. J. (2008). A crucial role in cell spreading for the interaction of Abl PxxP motifs with Crk and Nck adaptors. *J. Cell Sci.* 121, 3071-3082. 10.1242/jcs.031575 18768933 PMC2768557

[BIO029900C2] Batlle E., Bacani J., Begthel H., Jonkeer S., Gregorieff A., van de Born M., Malats N., Sancho E., Boon E., Pawson T. et al. (2005). EphB receptor activity suppresses colorectal cancer progression. *Nature* 435, 1126-1130. 10.1038/nature03626 15973414

[BIO029900C3] Bouvier D., Corera A. T., Tremblay M.-E., Riad M., Chagnon M., Murai K. K., Pasquale E. B., Fon E. A. and Doucet G. (2008). Pre-synaptic and post-synaptic localization of EphA4 and EphB2 in adult mouse forebrain. *J. Neurochem.* 106, 682-695. 10.1111/j.1471-4159.2008.05416.x 18410519

[BIO029900C4] Boyd A. W., Bartlett P. F. and Lackmann M. (2014). Therapeutic targeting of EPH receptors and their ligands. *Nat. Rev. Drug Discov.* 13, 39-62. 10.1038/nrd4175 24378802

[BIO029900C5] Boyle S. N., Michaud G. A., Schweitzer B., Predki P. F. and Koleske A. J. (2007). A critical role for cortactin phosphorylation by abl-family kinases in PDGF-induced dorsal-wave formation. *Curr. Biol.* 17, 445-451. 10.1016/j.cub.2007.01.057 17306540

[BIO029900C6] Bugaj L. J., Choksi A. T., Mesuda C. K., Kane R. S. and Schaffer D. V. (2013). Optogenetic protein clustering and signaling activation in mammalian cells. *Nat. Methods* 10, 249-252. 10.1038/nmeth.2360 23377377

[BIO029900C7] Carter N., Nakamoto T., Hirai H. and Hunter T. (2002). EphrinA1-induced cytoskeletal re-organization requires FAK and p130(cas). *Nat. Cell Biol.* 4, 565-573. 10.1038/ncb823 12134157

[BIO029900C8] Chang K.-Y., Woo D., Jung H., Lee S., Kim S., Won J., Kyung T., Park H., Kim N., Yang H. W. et al. (2014). Light-inducible receptor tyrosine kinases that regulate neurotrophin signalling. *Nat. Commun.* 5, 4057 10.1038/ncomms5057 24894073

[BIO029900C9] Chen Y., Fu A. K. Y. and Ip N. Y. (2012). Eph receptors at synapses: Implications in neurodegenerative diseases. *Cell. Signal.* 24, 606-611. 10.1016/j.cellsig.2011.11.016 22120527

[BIO029900C10] Dai D., Huang Q., Nussinov R. and Ma B. (2014). Promiscuous and specific recognition among ephrins and Eph receptors. *Biochim. Biophys. Acta BBA - Proteins Proteomics* 1844, 1729-1740. 10.1016/j.bbapap.2014.07.002 25017878 PMC4157952

[BIO029900C11] Davis S., Gale N. W., Aldrich T. H., Maisonpierre P. C., Lhotak V., Pawson T., Goldfarb M. and Yancopoulos G. D. (1994). Ligands for EPH-related receptor tyrosine kinases that require membrane attachment or clustering for activity. *Science* 266, 816-819. 10.1126/science.7973638 7973638

[BIO029900C12] Ditlev J. A., Michalski P. J., Huber G., Rivera G. M., Mohler W. A., Loew L. M. and Mayer B. J. (2012). Stoichiometry of Nck-dependent actin polymerization in living cells. *J. Cell Biol.* 197, 643-658. 10.1083/jcb.201111113 22613834 PMC3365498

[BIO029900C13] Elowe S., Holland S. J., Kulkarni S. and Pawson T. (2001). Downregulation of the ras–mitogen-activated protein kinase pathway by the EphB2 receptor tyrosine kinase is required for ephrin-induced neurite retraction. *Mol. Cell. Biol.* 21, 7429-7441. 10.1128/MCB.21.21.7429-7441.2001 11585923 PMC99915

[BIO029900C14] Fang W. B., Brantley-Sieders D. M., Parker M. A., Reith A. D. and Chen J. (2005). A kinase-dependent role for EphA2 receptor in promoting tumor growth and metastasis. *Oncogene* 24, 7859-7868. 10.1038/sj.onc.1208937 16103880

[BIO029900C15] Genander M., Halford M. M., Xu N.-J., Eriksson M., Yu Z., Qiu Z., Martling A., Greicius G., Thakar S., Catchpole T. et al. (2009). Dissociation of EphB2 Signaling Pathways Mediating Progenitor Cell Proliferation and Tumor Suppression. *Cell* 139, 679-692. 10.1016/j.cell.2009.08.048 19914164 PMC2786256

[BIO029900C16] Guo Z. V., Hart A. C. and Ramanathan S. (2009). Optical interrogation of neural circuits in Caenorhabditis elegans. *Nat. Methods* 6, 891-896. 10.1038/nmeth.1397 19898486 PMC3108858

[BIO029900C17] Henkemeyer M., Itkis O. S., Ngo M., Hickmott P. W. and Ethell I. M. (2003). Multiple EphB receptor tyrosine kinases shape dendritic spines in the hippocampus. *J. Cell Biol.* 163, 1313-1326. 10.1083/jcb.200306033 14691139 PMC1435730

[BIO029900C18] Himanen J.-P., Chumley M. J., Lackmann M., Li C., Barton W. A., Jeffrey P. D., Vearing C., Geleick D., Feldheim D. A., Boyd A. W. et al. (2004). Repelling class discrimination: ephrin-A5 binds to and activates EphB2 receptor signaling. *Nat. Neurosci.* 7, 501-509. 10.1038/nn1237 15107857

[BIO029900C19] Himanen J. P., Yermekbayeva L., Janes P. W., Walker J. R., Xu K., Atapattu L., Rajashankar K. R., Mensinga A., Lackmann M., Nikolov D. B. et al. (2010). Architecture of Eph receptor clusters. *Proc. Natl. Acad. Sci. USA* 107, 10860-10865. 10.1073/pnas.1004148107 20505120 PMC2890748

[BIO029900C20] Irie F. and Yamaguchi Y. (2002). EphB receptors regulate dendritic spine development via intersectin, Cdc42 and N-WASP. *Nat. Neurosci.* 5, 1117-1118. 10.1038/nn964 12389031

[BIO029900C21] Janes P. W., Griesshaber B., Atapattu L., Nievergall E., Hii L. L., Mensinga A., Chheang C., Day B. W., Boyd A. W., Bastiaens P. I. et al. (2011). Eph receptor function is modulated by heterooligomerization of A and B type Eph receptors. *J. Cell Biol.* 195, 1033-1045. 10.1083/jcb.201104037 22144690 PMC3241718

[BIO029900C22] Janes P. W., Nievergall E. and Lackmann M. (2012). Concepts and consequences of Eph receptor clustering. *Semin. Cell Dev. Biol.* 23, 43-50. 10.1016/j.semcdb.2012.01.001 22261642

[BIO029900C23] Kayser M. S., Nolt M. J. and Dalva M. B. (2008). EphB receptors couple dendritic filopodia motility to synapse formation. *Neuron* 59, 56-69. 10.1016/j.neuron.2008.05.007 18614029 PMC2617787

[BIO029900C24] Kennedy M. J., Hughes R. M., Peteya L. A., Schwartz J. W., Ehlers M. D. and Tucker C. L. (2010). Rapid blue-light-mediated induction of protein interactions in living cells. *Nat. Methods* 7, 973-975. 10.1038/nmeth.1524 21037589 PMC3059133

[BIO029900C25] Kim N., Kim J. M., Lee M., Kim C. Y., Chang K.-Y. and Heo W. D. (2014). Spatiotemporal control of fibroblast growth factor receptor signals by blue light. *Chem. Biol.* 21, 903-912. 10.1016/j.chembiol.2014.05.013 24981772

[BIO029900C26] Korobova F. and Svitkina T. (2010). Molecular architecture of synaptic actin cytoskeleton in hippocampal neurons reveals a mechanism of dendritic spine morphogenesis. *Mol. Biol. Cell* 21, 165-176. 10.1091/mbc.E09-07-0596 19889835 PMC2801710

[BIO029900C27] Kullander K. and Klein R. (2002). Mechanisms and functions of eph and ephrin signalling. *Nat. Rev. Mol. Cell Biol.* 3, 475-486. 10.1038/nrm856 12094214

[BIO029900C28] Lapetina S., Mader C. C., Machida K., Mayer B. J. and Koleske A. J. (2009). Arg interacts with cortactin to promote adhesion-dependent cell edge protrusion. *J. Cell Biol.* 185, 503-519. 10.1083/jcb.200809085 19414610 PMC2700396

[BIO029900C29] Lin K.-T., Sloniowski S., Ethell D. W. and Ethell I. M. (2008). Ephrin-B2-induced cleavage of EphB2 receptor is mediated by matrix metalloproteinases to trigger cell repulsion. *J. Biol. Chem.* 283, 28969-28979. 10.1074/jbc.M804401200 18713744 PMC2570862

[BIO029900C30] Lin Y.-C., Yeckel M. F. and Koleske A. J. (2013). Abl2/Arg controls dendritic spine and dendrite arbor stability via distinct cytoskeletal control pathways. *J. Neurosci. Off. J. Soc. Neurosci.* 33, 1846-1857. 10.1523/JNEUROSCI.4284-12.2013 PMC3711664 23365224

[BIO029900C31] Lisabeth E. M., Falivelli G. and Pasquale E. B. (2013). Eph receptor signaling and ephrins. *Cold Spring Harb. Perspect. Biol.* 5, a009159 10.1101/cshperspect.a009159 24003208 PMC3753714

[BIO029900C32] Machida K., Thompson C. M., Dierck K., Jablonowski K., Kärkkäinen S., Liu B., Zhang H., Nash P. D., Newman D. K., Nollau P. et al. (2007). High-throughput phosphotyrosine profiling using SH2 domains. *Mol. Cell* 26, 899-915. 10.1016/j.molcel.2007.05.031 17588523

[BIO029900C33] Miao H., Burnett E., Kinch M., Simon E. and Wang B. (2000). Activation of EphA2 kinase suppresses integrin function and causes focal-adhesion-kinase dephosphorylation. *Nat. Cell Biol.* 2, 62-69. 10.1038/35000008 10655584

[BIO029900C34] Miao H., Wei B.-R., Peehl D. M., Li Q., Alexandrou T., Schelling J. R., Rhim J. S., Sedor J. R., Burnett E. and Wang B. (2001). Activation of EphA receptor tyrosine kinase inhibits the Ras/MAPK pathway. *Nat. Cell Biol.* 3, 527-530. 10.1038/35074604 11331884

[BIO029900C35] Moeller M. L., Shi Y., Reichardt L. F. and Ethell I. M. (2006). EphB receptors regulate dendritic spine morphogenesis through the recruitment/phosphorylation of focal adhesion kinase and RhoA activation. *J. Biol. Chem.* 281, 1587-1598. 10.1074/jbc.M511756200 16298995

[BIO029900C36] Noberini R., Rubio de la Torre E. and Pasquale E. B. (2012). Profiling Eph receptor expression in cells and tissues. *Cell Adhes. Migr.* 6, 102-156. 10.4161/cam.19620 PMC3499309 22568954

[BIO029900C37] Pabbisetty K. B., Yue X., Li C., Himanen J.-P., Zhou R., Nikolov D. B. and Hu L. (2007). Kinetic analysis of the binding of monomeric and dimeric ephrins to Eph receptors: Correlation to function in a growth cone collapse assay. *Protein Sci.* 16, 355-361. 10.1110/ps.062608807 17322526 PMC2203307

[BIO029900C38] Pasquale E. B. (2010). Eph receptors and ephrins in cancer: bidirectional signaling and beyond. *Nat. Rev. Cancer* 10, 165-180. 10.1038/nrc2806 20179713 PMC2921274

[BIO029900C39] Penzes P., Beeser A., Chernoff J., Schiller M. R., Eipper B. A., Mains R. E. and Huganir R. L. (2003). Rapid induction of dendritic spine morphogenesis by trans-synaptic ephrinB-EphB receptor activation of the Rho-GEF kalirin. *Neuron* 37, 263-274. 10.1016/S0896-6273(02)01168-6 12546821

[BIO029900C40] Schaupp A., Sabet O., Dudanova I., Ponserre M., Bastiaens P. and Klein R. (2014). The composition of EphB2 clusters determines the strength in the cellular repulsion response. *J. Cell Biol.* 204, 409-422. 10.1083/jcb.201305037 24469634 PMC3912530

[BIO029900C41] Seiradake E., Harlos K., Sutton G., Aricescu A. R. and Jones E. Y. (2010). An extracellular steric seeding mechanism for Eph-ephrin signaling platform assembly. *Nat. Struct. Mol. Biol.* 17, 398-402. 10.1038/nsmb.1782 20228801 PMC3672960

[BIO029900C42] Sloniowski S. and Ethell I. M. (2012). Looking forward to EphB signaling in synapses. *Semin. Cell Dev. Biol.* 23, 75-82. 10.1016/j.semcdb.2011.10.020 22040917 PMC3646480

[BIO029900C43] Stein E., Lane A. A., Cerretti D. P., Schoecklmann H. O., Schroff A. D., Van Etten R. L. and Daniel T. O. (1998). Eph receptors discriminate specific ligand oligomers to determine alternative signaling complexes, attachment, and assembly responses. *Genes Dev.* 12, 667-678. 10.1101/gad.12.5.667 9499402 PMC316584

[BIO029900C44] Taslimi A., Vrana J. D., Chen D., Borinskaya S., Mayer B. J., Kennedy M. J. and Tucker C. L. (2014). An optimized optogenetic clustering tool for probing protein interaction and function. *Nat. Commun.* 5, 4925 10.1038/ncomms5925 25233328 PMC4170572

[BIO029900C45] Tatavarty V., Das S. and Yu J. (2012). Polarization of actin cytoskeleton is reduced in dendritic protrusions during early spine development in hippocampal neuron. *Mol. Biol. Cell* 23, 3167-3177. 10.1091/mbc.E12-02-0165 22740628 PMC3418311

[BIO029900C46] Zimmer M., Palmer A., Köhler J. and Klein R. (2003). EphB–ephrinB bi-directional endocytosis terminates adhesion allowing contact mediated repulsion. *Nat. Cell Biol.* 5, 869-878. 10.1038/ncb1045 12973358

[BIO029900C47] Ziv N. E. and Smith S. J. (1996). Evidence for a role of dendritic filopodia in synaptogenesis and spine formation. *Neuron* 17, 91-102. 10.1016/S0896-6273(00)80283-4 8755481

[BIO029900C48] Zou J. X., Wang B., Kalo M. S., Zisch A. H., Pasquale E. B. and Ruoslahti E. (1999). An Eph receptor regulates integrin activity through R-Ras. *Proc. Natl. Acad. Sci.* 96, 13813-13818. 10.1073/pnas.96.24.13813 10570155 PMC24147

