# Correction: Optogenetic activation of EphB2 receptor in dendrites induced actin polymerization by activating Arg kinase

**DOI:** 10.1242/bio.034694

**Published:** 2018-04-15

**Authors:** Clifford Locke, Kazuya Machida, Chandra L. Tucker, Yi Wu, Ji Yu

There was an error published in *Biology Open* (2017) **6**, 1820-1830; doi: 10.1242/bio.029900.

The author list was incomplete, and the text highlighted in bold below has been changed in the author list, author affiliations, author contributions and funding sections:

Clifford Locke^1^, Kazuya Machida^1^, **Chandra L. Tucker^2^,** Yi Wu^1^ and Ji Yu^1,*^

^1^Richard D. Berlin Center for Cell Analysis and Modeling, University of Connecticut Health Center, Farmington, CT 06030, USA. **^2^Department of Pharmacology, University of Colorado School of Medicine, Aurora, CO 80045, USA.**

*Author for correspondence (jyu@uchc.edu)

**Author contributions**

Conceptualization: Y.W., **C.L.T.,** J.Y.; Methodology: C.L.; Formal analysis: C.L., K.M.; Investigation: C.L., K.M.; Writing - original draft: C.L., J.Y.; Writing - review & editing: **C.L.T.,** J.Y.; Supervision: Y.W., J.Y.; Funding acquisition: J.Y.

**Funding**

This project was funded by National Institute of General Medical Sciences, grant no**s** GM123784, **GM117061** **and GM100225**, and by National Cancer Institute, grant no. CA154966.

A sentence in the Materials and Methods, ‘Reagents and cell culture’ section has also been corrected to:

OptoEphB2 as well as all variants and control constructs were derived from CRY2olig-mCherry or CRY2oligPHR-mCherry plasmids (Kennedy et al., 2010; Taslimi et al., 2014).

Biology Open and the authors apologise to the readers for any confusion that this omission might have caused.

