# Supplementary material for: Optogenetic activation of EphB2 receptor in dendrites induced actin polymerization by activating Arg kinase
Source: Biol Open. 2017 Nov 20;6(12):1820–30. doi: 10.1242/bio.029900 (PMC5769660; doi:10.1242/bio.029900)
Supplement: Supplementary information [file biolopen-6-029900-s1.pdf]

Supplementary Figures

```
MGSSKSKPKLCNRRGFERADSEYTDKLQHYTSGHMTPGMKIYIDPFTYEDPNEAVREFAKEIDI
SCVKIEQVIGAGEFGEVCSGHLKLPGKREIFVAIKTLKSGYTEKQRRDFLSEASIMGQFDHPNV
IHLEGVVTKSTPVMIITEFMENGSLDSFLRQNDGQFTVIQLVGMLRGIAAGMKYLADMNYVHRD
LAARNILVNSNLVCKVSDFGLSRFLEDDTSDPTYTSALGGKIPIRWTAPEAIQYRKFTSASDVW
SYGIVMWEVMSYGERPYWDMTNQDVINAIEQDYRLPPPMDCPSALHQLMLDCWQKDRNHRPKFG
QIVNTLDKMIRNPNSLKAMAPLSSGINLPLLDRTIPDYTSFNTVDEWLEAIKMGQYKESFANAG
FTSFDVVSQMMMEDILRVGVTLAGHQKKILNSIQVMRAQMNQIQSVEVWRADPAFLYKVVRFEA
TMKMDKKTIVWFRRDLRIEDNPALAAAAHEGSVFPVFIWCPEEEGQFYPGRASRWWMKQSLAHL
SQSLKALGSDLTLIKTHNTISAILDCIRVTGATKVVFNHLYDPVSLVRDHTVKEKLVERGISVQ
SYNGDLLYEPWEIYCEKGKPFTSFNSYWKKCLDMSIESVMLPPPWRLMPITAAAEAIWACSIEE
LGLENEAEKPSNALLTRAWSPGWSNADKLLNEFIEKQLIDYAKNSKKVVGNSTSLLSPYLHFGE
ISVRHVFQCARMKQIIWARDKNSEGEESADLFLRGIGLREYSRYICFNFPFTHEQSLLSHLRFF
PWDADVDKFKAWRQGRTGYPLVDAGMRELWATGWMHNRIRVIVSSFAVKFLLLPWKWGMKYFWD
TLLDADLECDILGWQYISGSIPDGHELDRLDNPALQGAKYDPEGEYIRQWLPELARLPTEWIHH
PWDAPLTVLKASGVELGTNYAKPIVDIDTARELLAKAISRTREAQIMIGAAARDPPVATMVSKG
EEDNMAIIKEFMRFKVHMEGSVNGHEFEIEGEGEGRPYEGTQTAKLKVTKGGPLPFAWDILSPQ
FMYGSKAYVKHPADIPDYLKLSFPEGFKWERVMNFEDGGVVTVTQDSSLQDGEFIYKVKLRGTN
FPSDGPVMQKKTMGWEASSERMYPEDGALKGEIKQRLKLKDGGHYDAEVKTTYKAKKPVQLPGA
YNVNIKLDITSHNEDYTIVEQYERAEGRHSTGGMDELYK
```

Color code:
Myristoylation signal peptide - EphB2(565-986) – linker - Cry2oligPHR - linker – mCherry

Fig S1. OptoEphB2 sequence.

Biology Open • Supplementary information

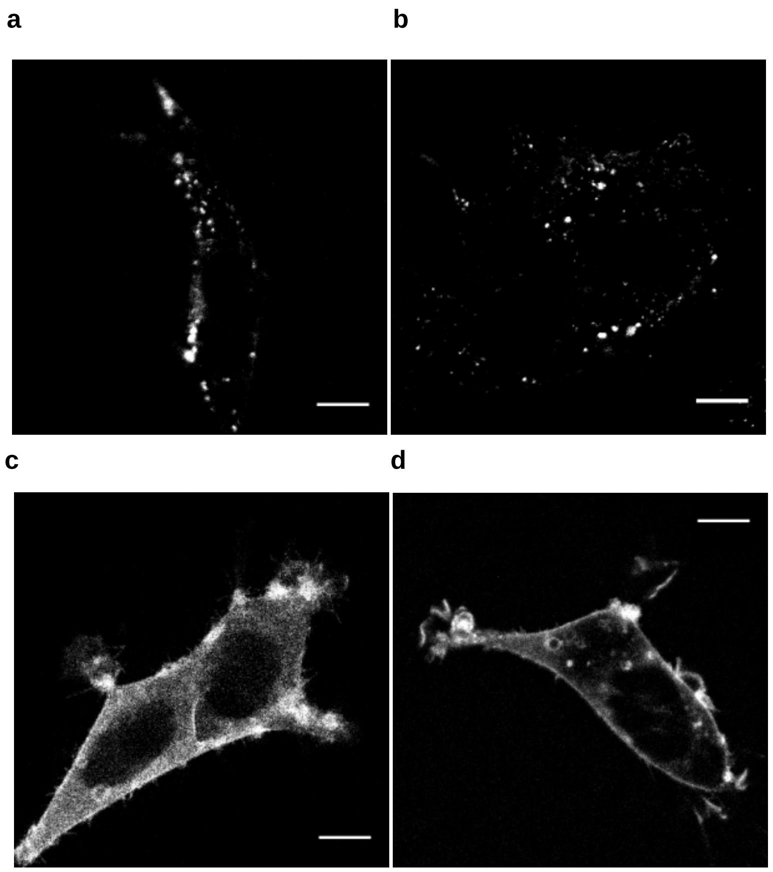

Figure S2. Confocal images of HEK293 cells expressing various Eph receptor fusion with Cry2PHR: (a) EphB1-Cry2PHR-mCherry, (b) EphB2-Cry2PHR-mCherry, (c) Myr-EphB2ICD-CryPHR-mCherry and (d) Myr-EphB2ICD-Cry2OligPHR-mCherry (optoEphB2). Expressing full-length EphB fusion with Cry2 resulted in internalization of the receptor. In comparison, optoEphB design resulted membrane localization. Scale bars, 10 µm.

Biology Open • Supplementary information

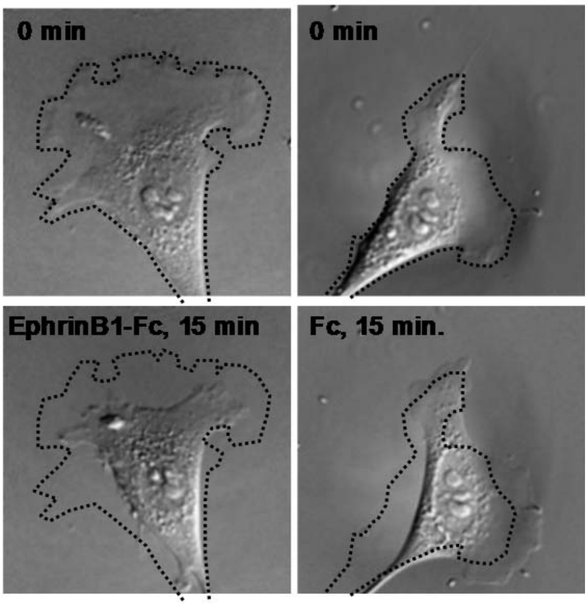

Figure S3. Ligand activation of EphB2 activation causes MEF cell rounding. Cells were stimulated with either human Fc (left column) or EphrinB1-Fc (right column), both preclustered with anti-Human-Fc. Dotted lines denote cell area before stimulation and solid line denote that after stimulation.

Biology Open • Supplementary information

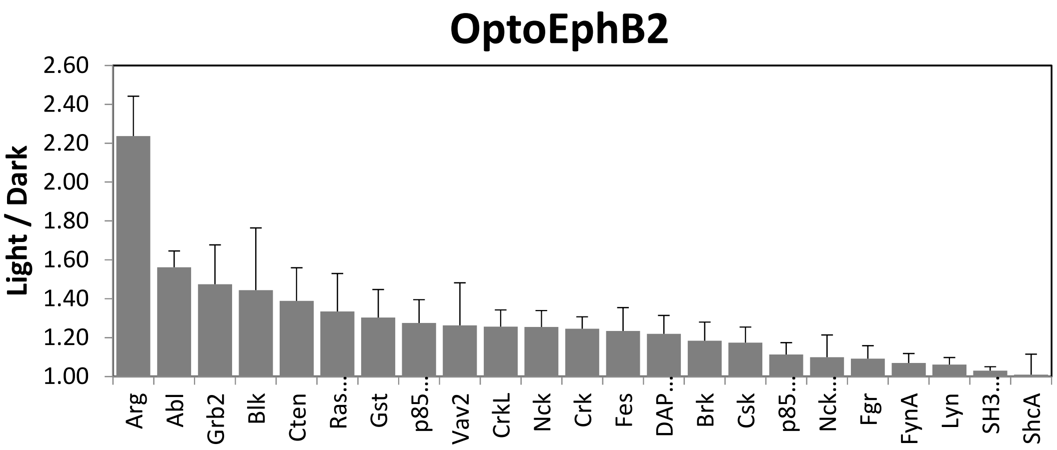

Figure S4. Summary of the Rosetta assay results on OptoEphB2 activation. Cell lysates were collected from MEF cells expressing OptoEphB2 either activated with blue light (1 min) or left in the dark. Only the probes showing induced binding were plotted.

Biology Open • Supplementary information

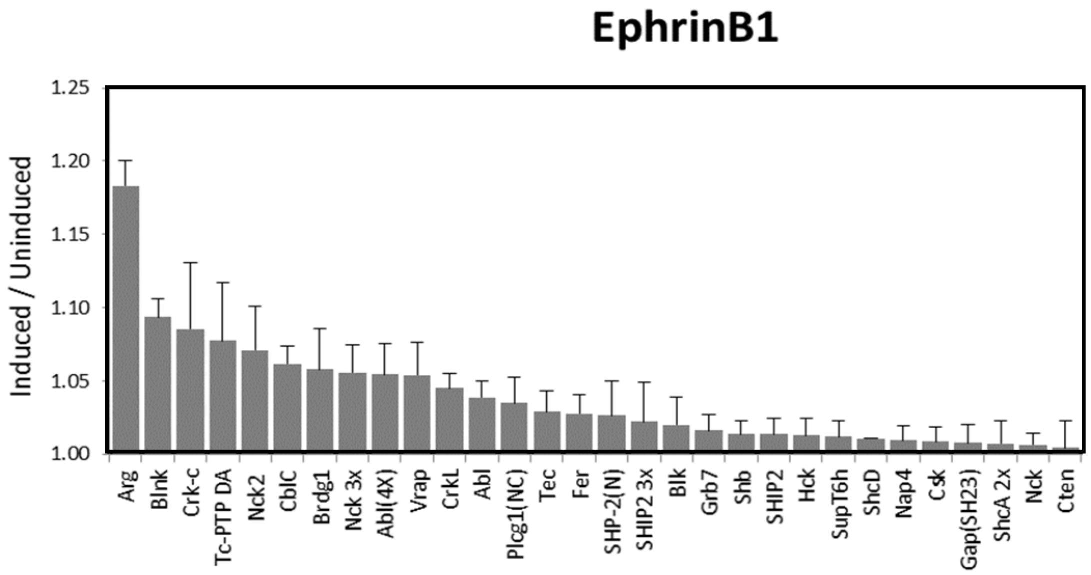

Figure S5. Summary of the Rosetta assay results on EphB2 activation using ephrinB1 ligand. Cell lysates were collected from EphB2-expressing MEF cells either treated with preclustered EphrinB1-Fc or Fc only. Only the probes showing induced binding were plotted.

Biology Open • Supplementary information

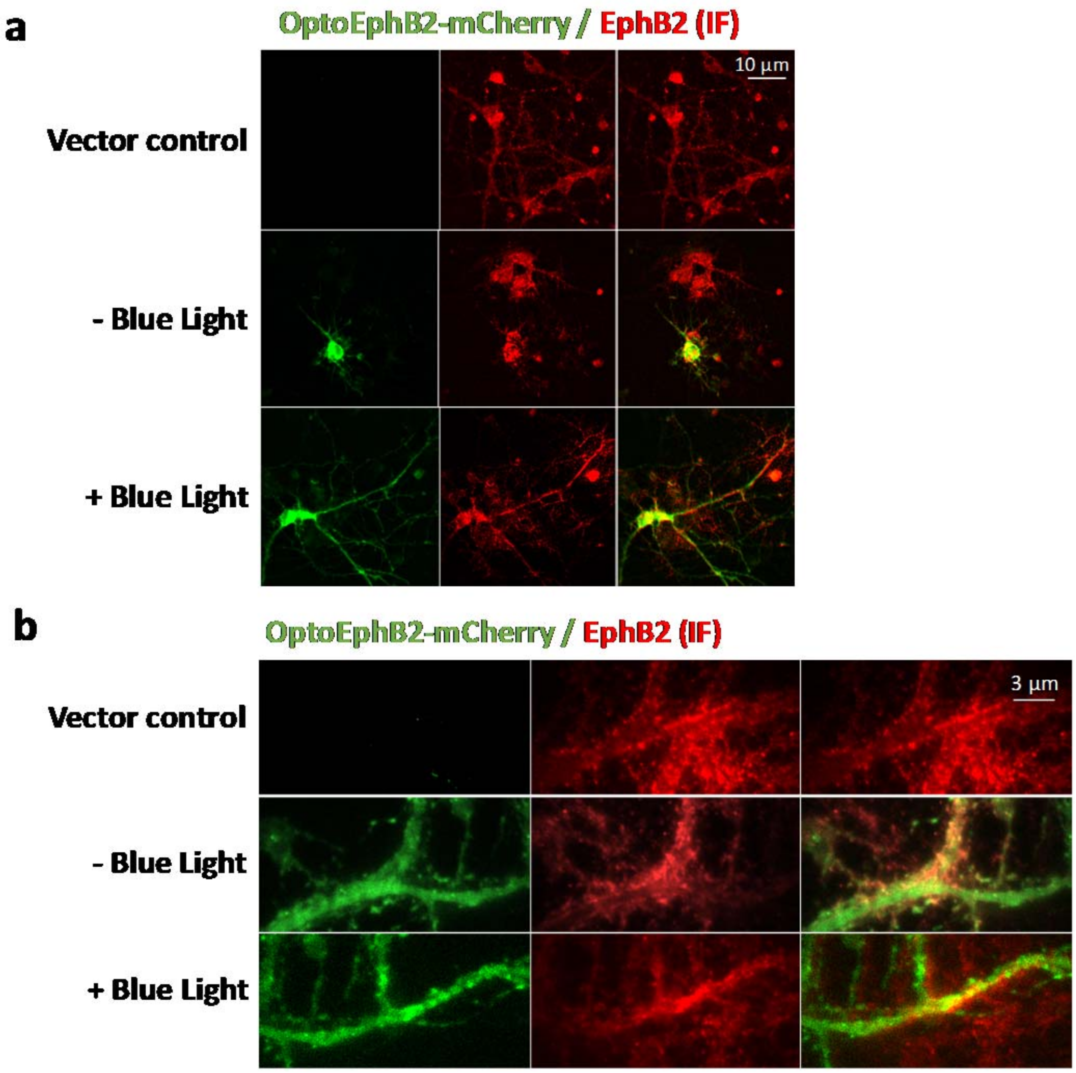

Figure S6. OptoEphB2 expression does not affect endogenous EphB2 localization. Cultured hippocampal neurons were transfected with either vector control or optoEphB2-mcherry on DIV10 and imaged on DIV11. Cells were fixed and endogenous EphB2 was immune-labeled (R&D systems AF467) with Cy5. Cells were imaged at either 20x (a) or 100x (b) magnification. EphB2 signal was found to be highest in the somadendritic compartment of neruons, but also in axons and glial cells to certain degree (a). At high magnification, membrane localization of EphB2 is evident in dendrites and EphB2 appears to be slightly clustered (b). No overt changes in EphB2 localization was detects in cells expressing optoEphB2 and cells activated with blue light.

Biology Open • Supplementary information

Movies

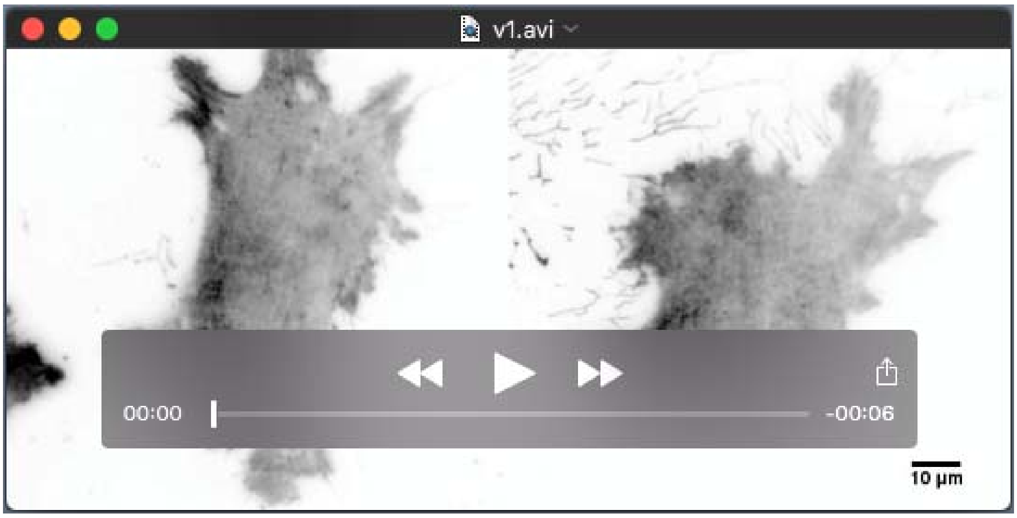

Video S1: Blue light illumination induce OptoEphB2 clustering in COS cells expressing either OptoEphB2-mCherry (left) or KD-optoEphB2-mCherry (right). Cell rounding was only observed in OptoEphB2-expressing cells. The blue dot denotes the time of blue light illumination.

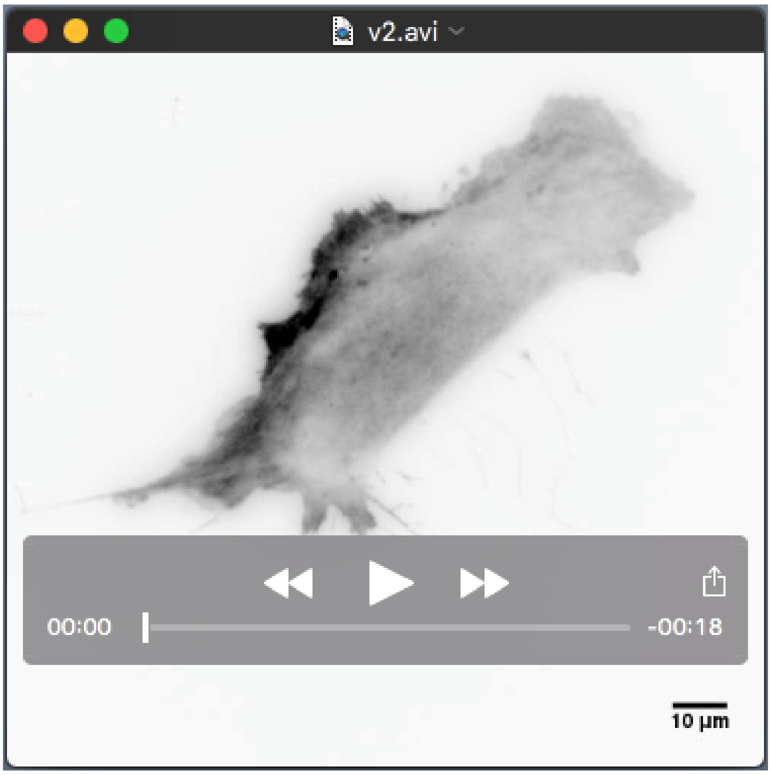

Video S2: Reversible OptoEphB2 clustering. MEF cells expressing OptoEphB2-mCherry exposed to periodical blue light illumination and followed via mCherry fluorescence. The blue dot denotes the time of blue light illumination.

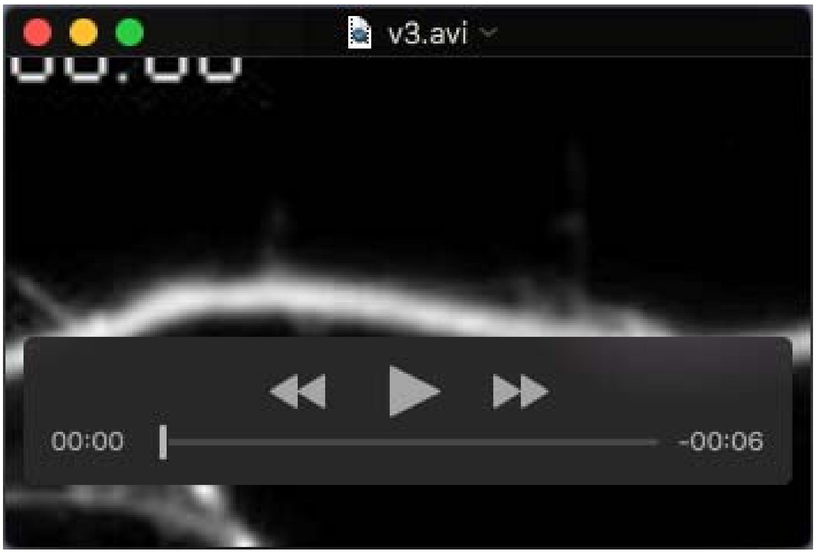

Video S3: OptoEphB2 activation induce filopodial protrusions in the dendrite. OptoEphB2 was locally photo-activated in the dendrite of cultured hippocampal neurons. The cell co-expressed OptoEphB2 and mCherry. Fluorescence signal is from mCherry. The blue circle denotes the area of blue light illumination. The blue dot denotes the time of blue light illumination.

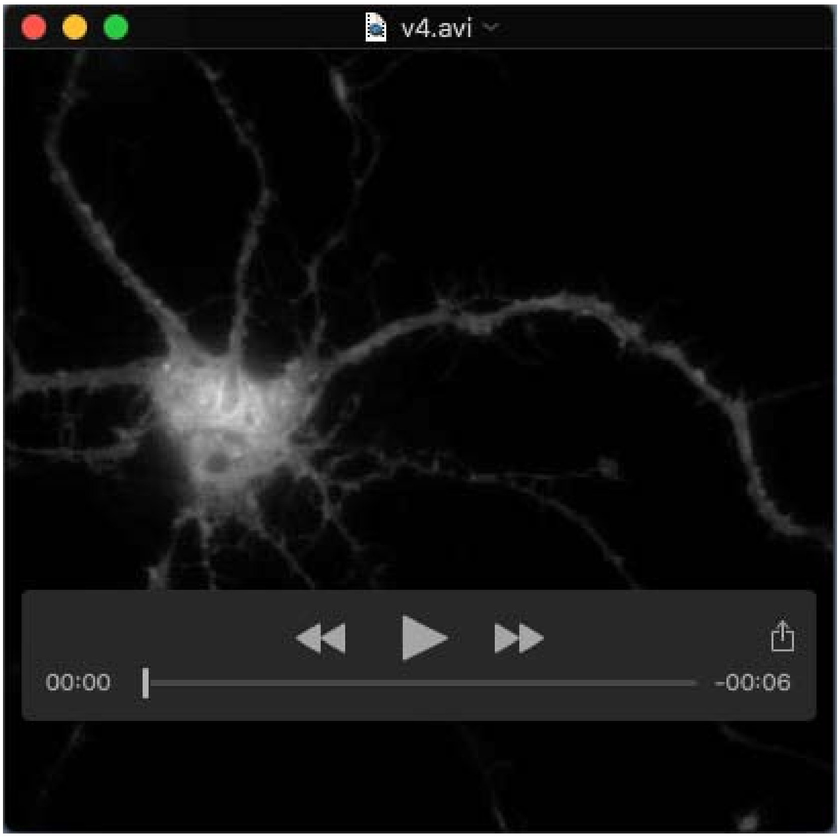

Video S4: KD-Arg inhibited OptoEphB2 induced dendritic filopodia. The video shows the OptoEphB2 fluorescence signal from a hippocampal neuron co-expressing KD-Arg and OptoEphB2. Blue light activation triggered OptoEphB2 clustering, but did not produce filopodial growth. Blue light was turned on at time 0:00 and remained on throughout the video. The blue circle denotes the area of blue light illumination.

Biology Open • Supplementary information
